# Impart: findings from a prison‐based model of HIV assisted partner notification in Indonesia

**DOI:** 10.1002/jia2.26132

**Published:** 2023-06-20

**Authors:** Gabriel J. Culbert, Judith A. Levy, Alana D. Steffen, Agung Waluyo, Valerie A. Earnshaw, Arie Rahadi

**Affiliations:** ^1^ Population Health Nursing Science College of Nursing, University of Illinois Chicago Chicago Illinois USA; ^2^ Health Policy & Administration School of Public Health, University of Illinois Chicago Chicago Illinois USA; ^3^ Faculty of Nursing Universitas Indonesia, Kota Depok Jawa Barat Indonesia; ^4^ Human Development and Family Sciences College of Education and Human Development, University of Delaware Newark Delaware USA; ^5^ AIDS Research Center Atma Jaya Catholic University Jakarta Indonesia

**Keywords:** contact tracing, HIV testing, Indonesia, partner notification, prisons, substance use

## Abstract

**Introduction:**

Assisted partner notification (APN) safely and effectively increases partner awareness of HIV exposure, testing and case identification in community settings. Nonetheless, it has not been specifically developed or evaluated for use in prison settings where people with HIV often are diagnosed and may have difficulty contacting or otherwise notifying partners. We developed Impart, a prison‐based APN model, and evaluated its efficacy in Indonesia to increase partner notification and HIV testing.

**Methods:**

From January 2020 to January 2021, 55 incarcerated men with HIV were recruited as index participants from six jail and prison facilities in Jakarta in a two‐group randomized trial comparing the outcomes of self‐tell notification (treatment as usual) versus Impart APN in increasing partner notification and HIV testing. Participants voluntarily provided names and contact information for sex and drug‐injection partners in the community with whom they had shared possible HIV exposure during the year prior to incarceration. Participants randomized to the self‐tell only condition were coached in how to notify their partners by phone, mail or during an in‐person visit within 6 weeks. Participants randomized to Impart APN could choose between self‐tell notification or anonymous APN by a two‐person team consisting of a nurse and outreach worker. We compared the proportion of partners in each group who were notified of exposure by the end of 6 weeks, subsequently tested and HIV diagnosed.

**Results:**

Index participants (*n* = 55) selected 117 partners for notification. Compared to self‐tell notification, Impart APN resulted in nearly a six‐fold increase in the odds of a named partner being notified of HIV exposure. Nearly two thirds of the partners notified through Impart APN (15/24) completed HIV testing within 6 weeks post notification compared to none of those whom participants had self‐notified. One‐third of the partners (5/15) who completed HIV testing post notification were diagnosed as HIV positive for the first time.

**Conclusions:**

Voluntary APN can be successfully implemented with a prison population and within a prison setting despite the many barriers to HIV notification that incarceration presents. Our findings suggest that the Impart model holds considerable promise to increase partner notification, HIV testing and diagnosis among sex and drug‐injecting partners of HIV‐positive incarcerated men.

## 1 INTRODUCTION

An estimated 3.8% of the world's prison population is HIV positive [1], and many of its inmates were first diagnosed and treated for HIV during incarceration. Although global HIV counselling guidelines stress the importance of encouraging individuals with HIV to inform their sex and drug‐injection partners of possible HIV exposure [2], prison inmates face numerous psychosocial and incarceration‐based challenges in doing so. Yet, most have former partners in the community who might benefit from learning of possible contact with the virus, HIV testing, and referral for treatment if needed.

With assisted partner notification (APN), health workers are trained to encourage and assist people diagnosed with HIV to inform their at‐risk partners of possible HIV exposure and the need for HIV testing. Research demonstrating APN's effectiveness in successfully promoting HIV disclosure to partners, HIV testing, diagnosis and treatment referral has accumulated for over three decades. Two early U.S. studies showed that APN was feasible in notifying sex and needle‐sharing partners of people diagnosed with HIV [3, 4]. Since then, APN has been replicated successfully in multiple settings globally and with differing populations, including clinic attendees in Malawi [5] and Kenya [6], people who inject drugs in Asia [7, 8], men who have sex with men in China [9] and cross‐border refugees in Uganda [10]. Research findings confirm that APN consistently yields higher rates of partner notification, HIV testing (1.5‐fold higher) and detection of new HIV cases (1.4‐fold higher) compared to self‐tell notification alone [11]. Even partners who choose not to test post‐notification stand to benefit from learning of contact with the virus as a possible prod towards protecting themselves in the future [12, 13]. Based on this growing body of research evidence, the World Health Organization has strongly endorsed training health workers to assist HIV‐positive individuals in notifying their at‐risk partners and including APN as a routine part of HIV services globally [14]. Nonetheless, its absence of mention in the scientific literature suggests that APN has never been introduced into prison settings or evaluated for its efficacy with incarcerated individuals.

Indonesia's incarcerated population is the eight largest globally, with more than a quarter million inmates [15]. Also, prisons in Indonesia historically constitute one of the main locations where people with HIV and those with substance use disorders are first offered HIV information, testing, diagnosis and treatment [16, 17]. HIV prevalence in Indonesia's prison population ranges from 1.2% to 6.5%, and is higher among women compared to men, and in large cities, such as Jakarta [18]. Per Indonesian guidelines, all new inmates are screened for HIV and offered antiretroviral therapy (ART) if needed. Meanwhile, Indonesian government statistics show that housewives with no known risk behaviour and a lack of personal awareness of being at HIV risk make up a sizable proportion of the country's new cases each year [19]. APN that reaches prison inmates’ wives and other sex partners to advise them of possible exposure offers an opportunity to identify some proportion of these women for HIV diagnosis and treatment at an earlier stage of the disease.

This research helps to affirm and assist the use of APN in prisons and other criminal justice facilities by implementing and comparing the efficacy of the Impart model of APN that we developed for prison populations versus sole reliance on prison inmates to inform their partners themselves (standard of care). With Impart APN, incarcerated people with HIV voluntarily identify and provide names and contact information for partners in the community with whom they may have shared HIV exposure before incarceration. They then select which of these partners to notify themselves or to have informed anonymously by an Impart two‐person team consisting of a nurse and community healthcare worker. Our study examines the outcome of each form of notification on how many at‐risk partners in the community are notified, their gender and type of risk exposure, and HIV testing outcomes. Its findings respond to the need for research on the effectiveness of APN programmes [20].

## 2 METHODS

### 2.1 Ethical statement

The Impart model and the procedures for this research were designed or selected in full recognition of the special ethical challenges of protecting the rights and safety of prisoners as a particularly vulnerable research population [21]. Informed consent was obtained from all HIV‐positive inmates both for their study participation and separately for each partner whom they gave researchers permission to notify. Informed consent was obtained from partners prior to HIV testing. All naming of partners was strictly voluntary, and no partners were notified without the index participant's permission. No compensation was given for agreeing to name or contact a partner; nor was there any type of penalty for declining to name or contact a partner. Prison medical staff who introduced the study to inmates had no role in selecting which inmates would join the study. Neither were prison staff involved in the study's data collection and partner elicitation procedures; nor did they have access to partner names or other personally identifiable study records. The research was approved by the Institutional Review Boards of the University of Illinois Chicago and Universitas Indonesia.

### 2.2 Study design

Our study population is comprised of HIV‐diagnosed men (index participants) receiving health services through HIV subspecialty clinics located within each jail and prison facility. A two‐group randomized trial was used to evaluate the effects of the prison‐based Impart APN model on partner notification and HIV testing outcomes. Index participants were allocated randomly to either to a self‐tell notification only standard of care condition or to Impart APN choice (active intervention) condition that allowed index participants to choose per partner between self‐tell or having an Impart team (consisting of an HIV‐trained nurse and outreach healthcare provider) notify the partner without revealing the identity of the index participant who named them. Approximately equal numbers of index participants were allocated to the intervention and control conditions per site, using block randomization to ensure balance in the two groups over time.

Our primary outcome was partner notification within 6 weeks as reported by the index participant or APN notifier. As secondary outcomes, we compared the number of partners in either group who by the end of this period had completed HIV testing including those who subsequently were HIV diagnosed. With permission from index participants, “self‐tell partners” in both study arms were contacted at the end of 6 weeks to verify if they had been notified and to obtain their HIV testing results. Those who had yet to be notified were informed of possible HIV exposure and offered HIV testing.

### 2.3 Sampling procedures

From January 2020 to January 2021, we recruited 55 incarcerated men with HIV from six all‐male jail and prison facilities in Jakarta, Indonesia representing differing security levels, population sizes and inmate characteristics. Prison medical staff introduced the study to HIV‐positive inmates using a project‐prepared script before or after their regularly scheduled clinic visits. Those inmates who indicated possible interest in participating were invited to meet privately with a research team member for screening and to learn more about the study prior to being asked to give informed consent.

To participate in the study, men had to be 18 years of age or older (by prison regulations), HIV positive (confirmed by HIV rapid test at enrolment), self‐reported as sexually active and/or injecting drugs during the year before incarceration, incarcerated within the last 3 years and more than 6 months away from their release date. Of the 241 inmates who were screened for possible participation, 175 were excluded from the study for having met one or more of its exclusion criteria. Of the 66 eligible inmates, 11 (15%) declined to participate due to feeling anxious or undecided about notifying partners (*n* = 7), lack of time or interest (*n* = 3) and illness (*n* = 1). Of the 66, 55 (83%) consented to participate.

### 2.4 Soliciting partner names and contact information

A project‐trained APN counsellor met with each inmate enrolled in the study to explain the importance of informing former sex and/or drug injection partners of possible HIV exposure and to encourage them to get tested and into treatment if needed. Each participant was asked to identify and provide contact information for those partners at HIV risk whom they might want to be notified. Index participants were allocated subsequently to one of the study's two conditions: self‐tell only (Indonesia's standard of care) or the Impart APN choice model, using a numbered sealed envelope system to conceal allocation from research staff [22].

### 2.5 Study conditions: self‐tell only notification versus Impart APN

#### 2.5.1 Self‐tell only notification

Participants randomized to self‐tell notification were assisted in developing a notification plan and coached in how to tell their at‐risk partners by the end of 6 weeks. They also were asked for permission to verify whom they had told or not when that period ended. Inmates could choose to tell their partner(s) by telephone, mail or in‐person during visitation at the jail/prison facility. They also could elect to have an HIV counsellor present during in‐person telling or by telephone to answer any clinical questions that the partner might have.

#### 2.5.2 Impart APN

Participants randomized to the Impart arm could choose per partner between sell‐tell notification or having an Impart APN‐trained nurse and community outreach healthcare provider work as a team to locate and notify their partners without revealing the identity of who had named them. With the latter choice, Impart notifiers initially contacted named partners by telephone and identified themselves as research‐affiliated health workers with important information about their health that needed to be shared in person. Partners who agreed to meet were asked to choose a location. Notification by telephone was delivered only as a last resort when circumstances prevented in‐person notification or a partner insisted on learning the reason for the telephone call. To locate partners whose contact information was unknown or could not be reached by telephone, APN notifiers visited the partner's residence, known “hang‐out spots” or other address(es) as necessary to find them. Notifiers were instructed never to reveal why they were searching for the person but only to say that they had some important information for them. Once contact was made at a private location, partners were advised of their possible shared exposure to HIV. To protect the confidentiality of incarcerated index participants, APN notifiers were given no information about the index participant and thus could not reveal their names or provide identifying information during this meeting.

#### 2.5.3 Partner HIV testing post notification

Partners notified by the Impart team were offered immediate HIV rapid testing using a fingerstick, combination antigen‐antibody test [23]. Alternatively, partners who preferred something other than point‐of‐contact testing or additional time to consider taking the test were referred to a clinic of their choice from a list of nearby free HIV services. Meanwhile, index participants randomized to the self‐tell condition and those assigned to Impart who chose to tell one or more partners themselves were given the same provider list to share with their partners in encouraging them to get HIV tested.

#### 2.5.4 Data collection

Socio‐demographic and health information were obtained from all HIV‐positive inmates enrolled as index participants prior to randomized group assignment. Using code numbers instead of personal identifiers, research staff recorded group assignment for each index participant, the number and characteristics of partners named (gender, approximate age and mode of exposure) and Impart participants’ preference per partner for self‐tell versus APN notification. Although no compensation was given for agreeing to name or contact partners, all participants received a snack and basic toiletry kit as thanks for their time in being interviewed, even if they chose not to name or contact partners.

Six weeks after the initial naming session, participants were reinterviewed about their notification experience. Participants in both arms who intended to inform one or more partners were asked to report on which (if any) they had informed. All self‐reported outcomes, including results from their verification, were entered into the project database to supplement information drawn from the logs that Impart notifiers kept recording the details and outcome of each successful and unsuccessful notification. After 6 weeks, APN was offered to notify any partner previously tried as self‐tell; however, those results are not reported here.

#### 2.5.5 Statistical analysis

Our primary analysis compared the study's two groups (self‐tell only to Impart assignment) on primary and secondary outcomes using intention‐to‐treat (ITT). All partners selected for notification were included in the analysis irrespective of group allocation or notification method. Partner notification outcomes were examined using logistic regression with robust standard errors to account for clustering due to partners having a shared index participant. Partner gender and partner type were selected beforehand as covariates.

For our primary analysis, partners with missing outcomes (*n* = 3) were treated as failed notifications. Sensitivity analyses (modified ITT and per protocol) compared outcomes for the two groups under different scenarios for missing data. To model a most‐conservative scenario for missing outcomes in the control group (modified ITT), we imputed successful notification outcomes (missing equals success) for two partners in the self‐tell notification (control) group and unsuccessful outcomes (missing equals failure) for one partner in the Impart APN group. A complete case analysis excluded these same three participants. In an as‐treated analysis, we compared successful notifications among partners selected for Impart APN with those selected for self‐tell notification whether by randomization or by choice.

## 3 RESULTS

### 3.1 Socio‐demographic characteristics of index participants (*n* = 55)

As shown in Table 1, the index participants’ median age was 36 years, and the median number of years since HIV diagnosis was 2.3 years. More than half (32/55) were HIV diagnosed during their current prison term. Inmates on ART before incarceration (21/55) reported poor adherence and uncertainty as to having been virally suppressed. Few reported consistent condom use or knowledge of their partner's HIV status. More than half the sample (32/55) reported sex with two or more partners in the 12 months prior to incarceration. About one‐third of participants (*n* = 17) also reported sharing injection equipment with a median of three drug‐injecting partners.

**Table 1:** Characteristics of incarcerated men with HIV recruited as index participants (*n* = 55)

**Characteristic**	**Median**	**IQR**
Median age in years (IQR)	36	32−40
Median years since HIV diagnosis (IQR)	2.3	0.8−6.6

	***n***	**%**
Diagnosed during current prison term	32	58.2
Diagnosed during a previous prison term	12	21.8
Diagnosed in the community before the current prison term	11	20.0
Receiving ART before incarceration	21	38.2
Receiving ART currently in jail or prison	47	85.5
Sexually active during the year before incarceration^a^	52	93.9
one sex partner	20	36.4
two sex partners	11	20.0
three sex partners	8	14.5
≥ four sex partners	13	23.6
Injecting drugs before incarceration	17	30.9
two needle‐sharing partners	6	−
three needle‐sharing partners	3	−
≥ four needle‐sharing partners	8	−

Abbreviations: ART, antiretroviral therapy; IQR, interquartile range.

^a^ Three participants reported only needle‐sharing partners.

### 3.2 Socio‐demographic characteristic of partners (117) identified for HIV notification

**Figure 1:**
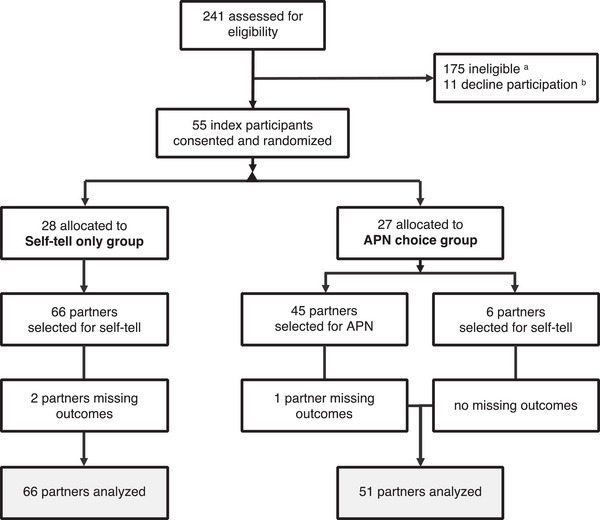
Study flow diagram. Abbreviation: APN, assisted partner notification ^a^ We excluded men (*n* = 175) who were incarcerated more than 3 years (42%) or within 6 months of their planned release date (8%). We also excluded men who were unable to provide names for partners (22%), had already notified all partners (21%), reported no partners during the contact tracing period (18%) or reported that their partners were deceased (11%) or residing outside Jakarta (9%). We excluded men (*n* = 175) who were: incarcerated more than 3 years (42%) or within 6 months of their planned release date (8%). We also excluded men who were unable to provide names for partners (22%), had already notified all partners (21%) or reported no partners during the contact tracing period (18%), or reported that their partners were deceased (11%) or residing outside Jakarta (9%). Some men were ineligible for more than one reason. ^b^ Most men who met the study's eligibility criteria (55/66) agreed to participate; however, 11 declined to participate for reasons that included: feeling anxious (*n* = 4) or undecided (*n* = 3), lack of time (*n* = 2) or interest (*n* = 1) and illness (*n* = 1). Collectively, the 55 index participants identified 117 partners for notification. Participants (*n* = 28) randomized to the self‐tell only condition identified 66 partners. Impart‐assigned participants (*n* = 27) identified 51 partners, of whom six were selected to be personally notified and 45 (88%) through Impart APN. Table 2 shows the characteristics of partners whom index participants chose to notify.

All partners selected for notification were included in the analysis irrespective of group allocation or notification method. Figure 1 shows a study flow diagram.

Partners ranged from 17 to 56 years of age (median of 28 years) and on average were younger than the index participants who named them. Most of those named (91/117) had been exposed through sex, including as spouses and regular sex partners, while 22% (26/117) had shared drug injection equipment. Most sex partners (*n* = 83/91) were female, whereas nearly all needle‐sharing partners (25/26) were male. Partner between‐group differences by gender and partner type were statistically significant (*p*<0.05).

### 3.3 HIV notification and testing outcomes

Table 3 shows partner notification and HIV testing outcomes by group and notification method. At the end of 6 weeks, index participants assigned to the self‐tell only condition reported having notified 18% (12/66) of their partners. Of these, five partners were contacted by researchers and two of them confirmed having been notified previously. In comparison, participants assigned to the Impart APN choice condition had personally notified 5/6 partners (two partners contacted and one confirmed), while 53% (24/45) had been notified through Impart APN.

**Table 2:** Characteristics of partners selected for notification (*n* = 117)

**Variable**	**Group allocation**			
	**Self‐tell notification group (*n* = 66)**		**APN choice group (*n* = 51)**	
Years of age (mean, SD)	29.2	7.3	29.9	6.7
Age range: 17–56 years				

	***n***	**%**	***n***	**%**
Gender				
female	54	81.8	29	56.9
male	12	18.2	22	43.1
Sex partner type				
main partner	14	21.2	23	45.1
regular sex partner	23	34.8	5	9.8
casual sex partner	19	28.2	6	11.8
Needle‐sharing partner	10	15.2	17	33.3
Notification method				
self‐tell	66	100.0	6	10.8
provider referral	−	−	45	88.2

Abbreviations: APN, assisted partner notification; SD, standard deviation.

**Table 3:** Partner notification and HIV testing outcomes after 6 weeks by group and notification method

	**Self‐tell notification group (*n* = 66)**	**APN choice group (*n* = 51)**	
*Notification method*	*Self‐tell (n = 66)*	*APN (n = 45)*	*Self‐tell (n = 6)*
**Notification outcome**			
Notified of HIV exposure	12^a^	24	5^b^
HIV tested	0^c^	15	0^c^
HIV diagnosed	0	5	0

^a^ Two partners confirmed as having been notified.

^b^ One partner confirmed as having been notified.

^c^ None of the self‐tell partners contacted by researchers reported having been tested for HIV.

The remaining 21 partners were unable to be located (*n* = 8), or deceased (*n* = 3), had become incarcerated themselves (*n* = 4), refused to meet (*n* = 2), resided outside Jakarta (*n* = 3), and one partner was not notified because the index participant withdrew from the study and asked to stop the notification. Nearly two thirds of partners notified by Impart APN (15/24) had completed HIV testing within six weeks compared to none of those whom participants had personally notified. Meanwhile, nine partners notified by Impart APN chose not to test because they refused further contact after notification (*n* = 3), were anxious about receiving their test results (*n* = 2), had recently been tested (*n* = 2) or already had been diagnosed with HIV (*n* = 2). Among partners who completed HIV testing, 33% (5/15) were newly HIV diagnosed. No adverse events were observed or reported during notification or follow‐up among index participants and partners in either group, despite careful probing for such events by researchers.

Table 4 reports the results of a multivariate, ITT analysis (*n* = 117) predicting the odds of partner notification occurring through each of the two notification conditions of the study. Compared to partners in the self‐tell only condition, partners designated for notification by inmates assigned to the Impart model were nearly six times more likely to be notified of exposure (unadjusted odds ratio = 5.93, 95% confidence interval [CI]: 2.82−12.47), *p*<0.001). Similar effect estimates were obtained from a model that adjusted for partner gender and partner type (adjusted odds ratio = 3.79, 95% CI: 1.61−8.92, *p* = 0.002).

**Table 4:** Multivariate models predicting partner notification from intention‐to‐treat analysis (*n* = 117)

**Variable**	**Unadjusted OR (95% CI)**	***p*‐value**	**Adjusted OR (95% CI)**	***p*‐value**
**Treatment arm**				
Self‐tell notification group	Reference		Reference	
APN choice group	5.93 (2.82−12.47)	<0.001	3.79 (1.61−8.92)	0.002
**Partner gender**				
Female	Reference		Reference	
Male	0.34 (0.17−0.68)	0.002	0.21 (0.07−0.74)	0.015
**Partner type**		<0.001		0.045
Main sex partner	Reference		Reference	
Regular/casual sex partner	0.22 (0.09−0.52)	0.001	0.31 (0.10−0.94)	0.038
Needle‐sharing partner	0.81 (0.34−1.93)	0.637	0.20 (0.05−0.82)	0.026

Note: All sensitivity analyses (Appendix S1) were consistent with a positive effect of Impart APN on partner notification.

Abbreviations: APN, assisted partner notification; CI, confidence interval; OR, odds ratio. Note: missing = failure.

## 4 DISCUSSION

Our research demonstrates that voluntary APN can be implemented successfully and ethically within the organizational structure of a prison setting. The Impart model's positive effects in increasing partner notification remained significant in and after adjusting for partner sex and type and even when assuming best‐case scenarios for missing verification data in the control group. The use of clinically trained notifier teams equipped to offer immediate point‐of‐contact HIV testing was successful to increase HIV testing and find new cases.

In general, the motivation to inform a partner of HIV exposure can be attributed to one or more factors, including self‐perception of ethical responsibility, concerns about the partner's health or to secure social support [24]. For whatever personal reason index participants in our study may have had, all together they named 117 partners. While three inmates assigned to the Impart arm elected to tell a partner themselves, APN notification was clearly the choice of most participants irrespective of transmission route or the relationship of the partner to the index person. This finding adds to a small but growing body of APN‐related research in other countries and in non‐prison settings reporting similar results [10, 13, 25, 26, 27]. Most likely, delegating notification responsibility to a third party alleviates the personal burden and difficulties associated with notifying a partner oneself.

No one approach to partner notification works best, and a combination of different approaches is needed [24]. Index participants assigned to the Impart APN arm were free to choose which partners to inform and how to tell them—two features of the model that permit the index person some formal control over the notification process. Of the 51 partners whom they named for notification, five were personally notified and the Impart team was able to find and notify 24 others. Meanwhile, of the 66 partners named by participants in the study's self‐tell arm, only 12 partners were reported as having been informed, while the others had not been contacted. We cannot tell from our data with scientific validity if all index participants in this arm ever seriously intended to personally tell all 66, merely named them without a personal commitment to do so, tried but were unsuccessful, or had developed second thoughts against informing them. Nonetheless, it is clear from our findings that Impart APN yielded a higher rate of partner notification than solely relying on index persons to do so.

Impart point‐of‐contact HIV testing also yielded a higher success rate than depending on an index person alone to encourage their partners to be tested. Of the 24 partners contacted in the Impart arm, 15 completed immediate point‐of‐contact HIV testing, of whom five were newly diagnosed and referred for treatment. Given that each partner contacted through APN was suspected or known to have been exposed to HIV, even partners who declined testing or who tested negative stood to benefit from notification counselling in HIV prevention.

### 4.1 Limitations of the study

Limitations of the study include relatively small sample size, short duration of follow‐up and possible bias due to incorrect self‐reporting of self‐tell outcomes. Nevertheless, to our knowledge, this research represents the first and largest study to develop and evaluate an APN model for use in prison settings. We are fully aware that fewer than half of the partners who index participants reported having personally told (7/17) could be confirmed. Yet, if all such reports of successful self‐tell notifications are indeed valid, the Impart choice model still proved three to four times more effective in successfully notifying at‐risk partners and generating HIV testing than self‐tell notification alone.

## 5 CONCLUSIONS

Our results confirm that the Impart APN model can be implemented successfully in jail and prison settings despite the many barriers to HIV notification that incarceration presents. With its trained team of notifiers, the model provides an effective means for HIV‐positive prisoners to inform HIV‐exposed partners whom they otherwise might not be able to reach or wish personally to tell. Although this study was conducted in Indonesia, the Impart model holds strong potential for successful implementation in other countries and with other incarcerated populations. It warrants further exploration.

## COMPETING INTERESTS

The authors declare no competing interests.

## AUTHORS’ CONTRIBUTIONS

All authors have contributed equally to the study's conceptualization and design. GJC obtained funding as Principal Investigator and wrote the initial manuscript with JL and AR. AW and AR supervised the research in Indonesia and analysed data with AS who planned the statistical analysis. VAE helped to develop the study's protocols and assessments and contributed with all authors to the interpretation of findings and revising the final manuscript.

## FUNDING

Research reported in this publication was supported by the National Institute of Mental Health of the National Institutes of Health under award number R34 MH115779 to GJC.

## Supporting information


**Appendix 1**. Multivariate models predicting partner notification from sensitivity analyses (n=117)

## Data Availability

Data requests referencing protocol# 2019‐0196 (PI: Culbert) may be sent to the Director of Research Facilitation at the University of Illinois Chicago College of Nursing.
