# Supplementary material for: Impart: findings from a prison‐based model of HIV assisted partner notification in Indonesia
Source: J Int AIDS Soc. 2023 Jun 20;26(6):e26132. doi: 10.1002/jia2.26132 (PMC10281637; doi:10.1002/jia2.26132)
Supplement: Supplementary file 1 — Appendix 1. Multivariate models predicting partner notification from sensitivity analyses (n=117) [file JIA2-26-e26132-s001.docx]

**Appendix 1**. Multivariate models predicting partner notification from sensitivity analyses (n=117)

|  | **Modified ITT analysis** (n=117)^a^ | | **Per-protocol analysis** (n=114)^b^ | | **As-treated analysis** (n=117)^c^ | |
| --- | --- | --- | --- | --- | --- | --- |
| **Variable** | **aOR (95% CI)** | **p-value** | **aOR (95% CI)** | **p-value** | **aOR (95% CI)** | **p-value** |
| Treatment Arm |  |  |  |  |  |  |
| Self-tell notification group | Reference |  | Reference |  | Reference |  |
| APN choice group | 3.00 (1.26-7.13) | 0.013 | 3.64 (1.51-8.79) | 0.004 | 2.35 (0.89-6.21) | 0.085 |
| Partner Gender |  |  |  |  |  |  |
| Female | Reference |  | Reference |  | Reference |  |
| Male | 0.12 (0.02-0.58) | 0.009 | 0.17 (0.04-0.72) | 0.016 | 0.16 (0.04-0.58) | 0.006 |
| Partner Type |  | 0.019 |  | 0.043 |  | 0.012 |
| Main sex partner | Reference |  | Reference |  | Reference |  |
| Regular/casual sex partner | 0.28 (0.09-0.85) | 0.025 | 0.32 (0.10-1.01) | 0.052 | 0.26 (0.08-0.81) | 0.020 |
| Needle-sharing partner | 0.10 (0.02-0.60) | 0.011 | 0.16 (0.03-0.76) | 0.021 | 0.15 (0.03-0.62) | 0.009 |
| aOR: Adjusted odds ratio; APN: Assisted partner notification; CI: Confidence interval; ITT: Intention to treat. | | | | | | |
| ^a^ Data imputed for two partners with missing outcomes in the self-tell notification group (missing=success) and one in the APN choice group (missing=failure) | | | | | | |
| ^b^ Excludes two partners in the self-tell notification group and one in the APN choice group with missing outcomes | | | | | | |
| ^c^ Compares partners selected for self-tell notification (by choice or randomization, n=72) to partners selected for APN (n=45) | | | | | | |
